# Hummingbird foraging preferences during extreme heat events

**DOI:** 10.1002/ece3.10053

**Published:** 2023-05-10

**Authors:** Sabina Lucke Lawrence, Jenny Hazlehurst

**Affiliations:** ^1^ Department of Biological Sciences California State University East Bay Hayward California USA

**Keywords:** extreme heat, foraging behavior, heat waves, hummingbirds, microsite preference

## Abstract

Climate change is projected to increase global mean annual temperatures as well as the frequency and intensity of extreme heat events. These changes are anticipated to alter the behavior of animals as they seek to thermoregulate in extreme heat. An important area of research is understanding how mutualistic interactions between animals and plants, such as pollination, will be affected by the cascading effects of extreme heat on animal foraging behavior. In this study, we used an experimental and observational approach to quantify the effects of extreme heat on hummingbird foraging preferences for nectar sources in shady versus sunny microsites. We also quantified pollen deposition using artificial stigmas at these sites to quantify potential cascading effects on plant reproduction. We hypothesized that hummingbirds would respond to extreme heat by preferentially foraging in shady microsites, and that this would reduce pollen deposition in sunny microsites on hot days. We found little support for this hypothesis, instead hummingbirds preferred to forage in sunny microsites regardless of ambient temperature. We also found weak evidence for higher pollen deposition in sunny microsites on hot days.

## Graphical abstract

Heat waves are predicted to get more intense under global climate change. We used experimental and observational data to determine if hummingbirds prefer to forage in shady microsites during heat waves, and how this might affect pollen deposition. Hummingbirds always preferred to forage in sunny microsites regardless of ambient temperature in both feeder trials and semi‐natural behavioral observation experiments, and there was no effect of ambient temperature or microsite on pollen deposition.

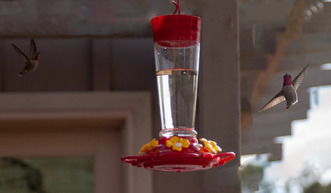

## 1 INTRODUCTION

The continuation of pollination services to plants in a warming climate is critical to sustaining plant biodiversity and ecosystem function. A meta‐analysis of plant dependence on vertebrate pollinators found that when birds were excluded from pollinating, fruit or seed production was reduced by 46% (Ratto et al., 2018). Changes in pollinator behavior can have cascading effects on plant populations (Anderson et al., 2011), as pollinator visitation rates have a positive effect on pollen receipt (Engel & Irwin, 2003). An estimated 87.5% of flowering plant species rely on animal pollination (Ollerton et al., 2011). While some research has investigated the effects of higher ambient temperatures on insect pollinators such as bumblebees, vertebrate pollinators have received relatively little attention. Hummingbirds (Aves: Trochilidae) are a critical group of vertebrate pollinators in the western hemisphere, and visit over 1300 species of plants from 100 different families in the Americas (del Coro Arizmendi & Rodríguez‐Flores, 2012).

Hummingbirds are highly reliant on daily nectar from plant mutualists due to their high metabolic rates (Cronk & Ojeda, 2008; González‐Gómez et al., 2011; Shankar et al., 2019), though they do also eat insects as a source of amino acids that are absent from nectar (Battey, 2019; Clark & Russell, 2012). Hummingbirds have a low energy storage capacity and high fixed metabolic costs, and thus are sensitive to daily fluctuations in metabolic costs and energy availability (González‐Gómez et al., 2011; Shankar et al., 2019; Shankar et al., 2020). On extremely hot days, some birds will alter their movement ecology to preferentially spend time in the shade, or they may spend more time engaging in heat dissipating behaviors such as panting or spreading their wings. These behavioral shifts may come at an opportunity cost. For example, male Southern yellow‐billed hornbills foraging on hot days panted more frequently and spent more time in thermal refugia, resulting in decreased foraging success and body mass losses (Van de Ven et al., 2019). Du Plessis et al. (2012) found that the foraging effort of Southern pied babblers was not affected by ambient temperature, but foraging efficiency was negatively affected.

Hummingbirds may use behavioral thermoregulation techniques to cope with high daytime temperatures (Powers et al., 2017). Hummingbirds in southeastern Arizona were found to use heat dissipation areas (HDAs) around the eyes, shoulders, and feet for passive cooling when a thermal gradient existed between the ambient temperature and the HDA surface temperature. Passive cooling is only effective if ambient temperature is below HDA temperature. Powers et al. (2017) found that the thermal gradient driving passive heat dissipation in five species of North American hummingbird disappears between an ambient temperature of 36 and 40°C. Powers (1992) found that *C. anna* approaches hyperthermia at temperatures of 37°C. When temperatures exceed 35°C, hummingbirds may need to use behavioral thermoregulation, for example by retreating to shady microsites, to balance their daily energy budgets (Powers et al., 2017). All five hummingbird species studied experienced long daytime periods when their thermal gradient disappeared. Even in the absence of a thermal gradient from their HDAs, broad‐billed hummingbirds maintained their mean body surface temperature near 38°C (Powers et al., 2017). This suggests some alternate cooling mechanism such as behavioral thermoregulation. Hummingbirds of some species decrease certain activities, like territorial defense behavior, past a threshold ambient temperature of 19.9°C (González‐Gómez et al., 2011). An organism's operative temperature includes the combined effects of radiative, convective, and conductive heat flux specific to that organism (Van de Ven et al., 2019). While ambient temperature is immutable until the weather changes, the operative temperature of an animal can be changed in several ways, including by seeking out increased or decreased insolation, increased or decreased wind exposure, and shelter from or exposure to precipitation. The difference between ambient and operative temperatures can be physiologically significant. For small avian species, operative temperature can vary by up to 15–20°C between shaded and sunny microsites within the same habitat, dramatically changing the physiological cost of thermoregulation (van de Ven et al., 2019). Due to hummingbirds' high surface area to volume ratio, they experience a greater increase in operative temperature than larger avian species when they move from a shaded microsite into a sunny one (Abdu et al., 2018). If hummingbirds use behavioral thermoregulation in a way that alters their foraging behavior, it could have potential for cascading effects on plant reproduction.

California is both a global floral biodiversity hotspot with many unique ornithophilous plants (Myers, 2000) and is expected to experience more extreme heat waves and increased temperatures in the future. Northern California has experienced some of the greatest temperature anomalies in daily maximum temperatures (Luo et al., 2017). Between 1950 and 2005, the San Francisco Bay Area average annual maximum temperature increased by 0.95°C, and is likely to see additional annual mean warming of 1.8°C (Ackerly et al., 2018) making it an ideal location to study how heat waves may alter hummingbird foraging behavior and have cascading effects on pollination. In California there is only one year‐round resident hummingbird throughout the state, the Anna's hummingbird (*Calypte anna*). The Anna's hummingbird has experienced an expansion of its geographic range northwards, which may be related to supplemental feeding at backyard feeders, introduced floral species such as *Eucalyptus*, and climate change (Battey, 2019; Clark & Russell, 2012; Greig et al., 2017). The adaptability of *C. anna* to land use change and climate change make it an ideal species in which to study the potential for cascading effects of behavioral changes due to climate change on pollination, as it is likely to be an abundant visitor of California's unique and threatened native plants in the future. In ornithophilous plants that Anna's hummingbirds might visit, pollen is usually deposited by the anthers onto the forehead or throat of the bird during a visit to a flower. That pollen is then transferred to another flower's stigma during pollination.

In this study, we test the hypothesis that Anna's hummingbirds (*Calypte anna*) would preferentially forage at experimental feeders in shady microsites versus sunny microsites on hot days (operative temperature in the sun >35°C) when compared to average days over the summer in California. We also test for differences in pollen deposition on artificial stigmas at feeders between sunny and shady microsites on hot days compared to average days. We hypothesized that pollen deposition would be greater at shady versus sunny microsites on hot days as compared to average days.

## 2 MATERIALS AND METHODS

### 2.1 Study organism

**FIGURE 1:**
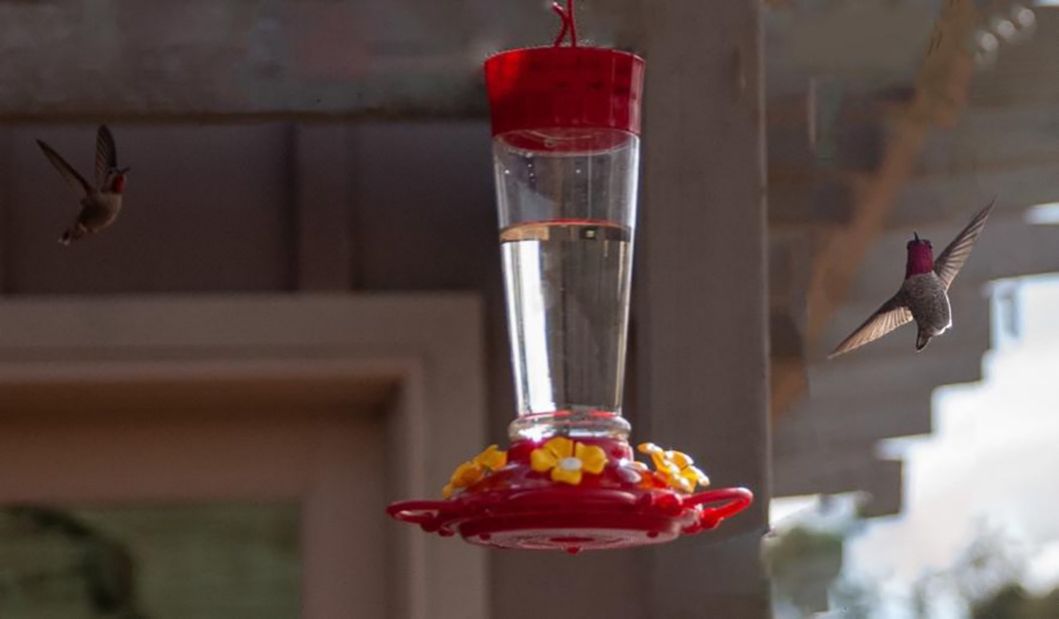
Anna's hummingbirds (*Calypte anna)* visiting a hummingbird feeder hanging on a porch in summer.

While most North American hummingbird diversity is concentrated in Mexico, approximately 24 species occur in the US and Canada. Eight hummingbird species occur in California as permanent or seasonal residents, with an additional six species recorded as rare vagrants. Anna's hummingbird (*Calypte anna*; Figure 1) are the only year‐round resident found throughout the state, and is the most commonly observed hummingbird in California. Anna's hummingbirds are on the larger side for hummingbirds in California, with a length of 10 cm and a weight of 3–6 g*. Calypte anna* uses a wide variety of habitats for foraging and nesting, including chaparral, oak and riparian woodlands, savannah, coastal scrub, and urban and suburban gardens (Clark & Russell, 2012). Some of the most frequently visited California native plant nectar sources include gooseberries (*Ribes*), monkeyflower (*Diplacus*), sage (*Salvia*), keckiellas (*Keckiella*), penstemons (*Penstemon*), and willowherb (*Epilobium*) (Clark & Russell, 2012). Most interactions between conspecifics are agonistic, including chases, vocalizations, and occasional contacts (Clark & Russell, 2012; González‐Gómez et al., 2011). Males defend breeding territories during the winter, and will attempt to mate with any female who enters the territory by courting her with dramatic display dives and advertising flights (Clark & Russell, 2012; Stiles, 1971). After the breeding season concludes in May, banding studies have revealed that many individuals migrate short distances to new feeding grounds either at a higher elevation or inland to the south or east, often returning to their original area after 2–3 months (Clark & Russell, 2012). However, because there are multiple patterns of local migration for *C. anna*, a site may have a year‐round hummingbird presence but substantial turnover of individuals (Clark & Russell, 2012).

### 2.2 Field site

**FIGURE 2:**
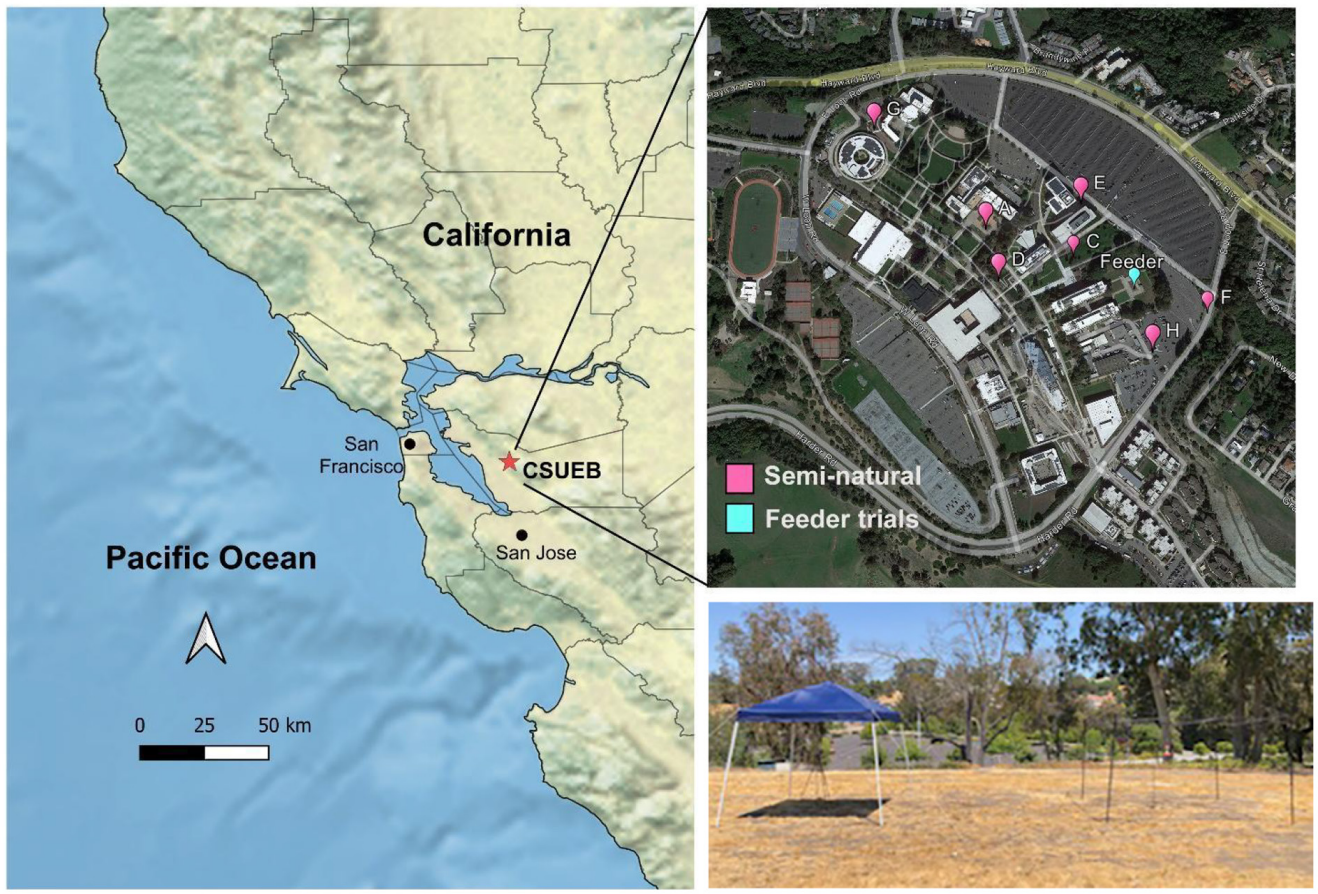
Locations of feeder trials (Feeder/Site B) and seminatural behavioral observations (Sites A, B, C, D, E, F, G, H) on the California State University East Bay (CSUEB) campus in Hayward, California. Inset shows Feeder Trial set up at Site B.

**FIGURE 3:**
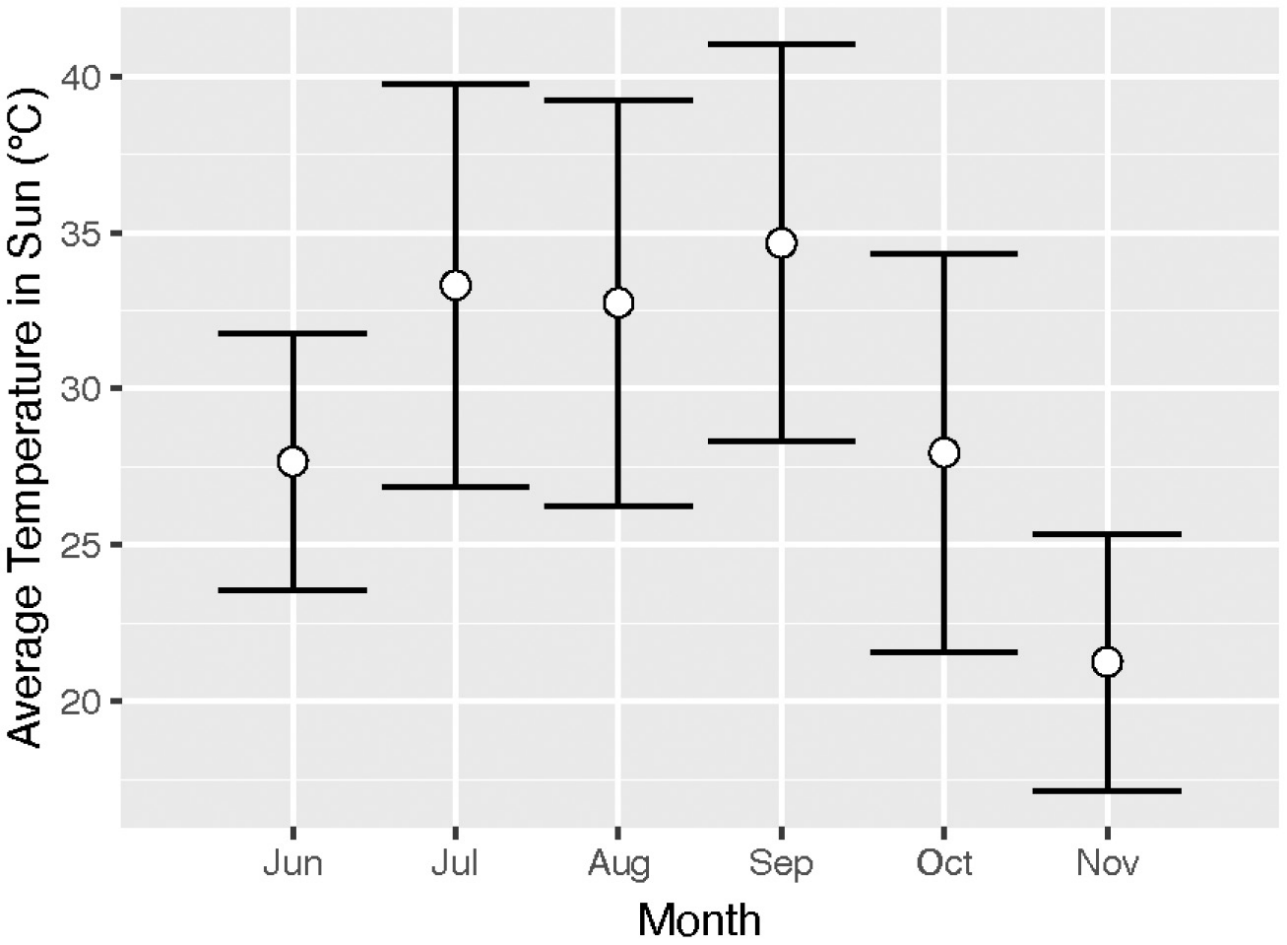
Average daily temperatures in sun at field site during months of feeder trials and seminatural observations. Error bars are standard deviations (SD).

This study took place in a seminatural environment on the California State University East Bay (CSUEB) campus in Hayward, California in the US (Figure 2). The study site consists of a mix of parking lots, buildings, paved walkways, and ornamental landscaping. The climate in the region is Mediterranean, with cool, mild winters and hot, dry summers, and the heat and dryness generally extend well into November. In the City of Hayward over the period 1991–2020, the summer average temperature was 18.9°C, summer high temperature was 23.9°C, and summer minimum temperature was 13.8°C, and the average humidity was 63%. This study took place from June 11, 2021–November 19, 2021, encompassing the hottest time of the year, and there was no precipitation during this time (Figure 3). Hot trials or hot observation sessions were categorized as when the maximum temperature in the sun was greater than 35°C.

### 2.3 Feeder trials

Glass feeders with five feeding ports and perches (Perky Pet, Inc.) containing a 30% sucrose solution were placed under a tent canopy with dimensions of 3.05 m × 3.05 m × 2.44 m (length × width  × height; Figure 2). Nectar concentrations in flowers visited by hummingbirds exhibit great natural variation, but average 25% sucrose (Pacini et al., 2003). Based on preliminary data from a low‐volume sucrose refractometer conducted during the period of this study, field sucrose concentrations at the study site ranged from 15 to 55%, and thus 30% is on the high end of average based on the literature, however, well within what a hummingbird might encounter at our field site. The sunny microsite treatment had the frame of the canopy, but not the shade cloth. The shady treatment had the shade cloth on and the feeder was within the shaded area for the duration of observations. The shade cloth of the canopy was made with 300D Polyester with a Polyurethane lining capable of withstanding 99% of ultraviolet rays. A single shade and a single sun treatment were placed 10 m apart in an open lawn to present foraging hummingbirds with a choice between the two microhabitats, and treatments were shuffled in position randomly during each session to avoid any spatial bias. Feeders were observed once per day between 12:30 p.m. and 5:30 p.m. to capture the hottest part of the day, after an acclimatization period of 1 h, and each observation session averaged 135 min (min = 111 min, max = 213 min, SD = 22 min). To track temperature in each microsite, iButton temperature sensors and dataloggers (Maxim Integrated, Inc.) were attached to the feeders using modeling clay. For each session the following environmental data were recorded: cloud cover (sunny, partly cloudy, overcast), observation start and end times, the number of people that walked within 25 m of the feeders during the observations (low: <10, moderate: 10–20, and high: >20), and the estimated number of open floral inflorescences within 25 m of the feeders. We quantified hummingbird preference by number of visits, defined as any time a bird entered the area under the canopy, foraging visits, defined as any time a hummingbird inserted its bill into the feeder, and visit duration, defined as the amount of time the bird spent in the canopy area.

### 2.4 Pollen deposition

We measured pollen deposition during feeder trials by placing artificial stigmas above the flowers on the feeding ports of the hummingbird feeders. Artificial stigmas were made by placing a 1 cm × 1 cm × 1 cm cube of fuchsin‐stained pollen collecting gelatin (Kearns & Inouye, 1993) inside a metal gemstone setting and attaching it to a length of wire 5 mm in length to simulate the stigma length and position of California fuchsia (*Epilobium canum*), a locally abundant hummingbird‐pollinated California native plant. At the end of a feeder trial, artificial stigmas were collected and mounted on glass slides for analysis. Slides were observed at a magnification of 100× using a digital microscope, and any pollen grains present were photographed. Pollen grains deposited per feeder trial were counted manually in slide photos using the program ImageJ (Schneider et al., 2012).

### 2.5 Seminatural behavioral observations

In order to provide context to our feeder experiments, we also conducted observations of free‐foraging birds in the campus landscape on extremely hot and average days during the same time period (11 June 2021–19 November 2021) as the feeder trials to determine if they preferred shady microsites to sunny microsites on extremely hot versus average days. Seminatural observations and feeder trials were both spread out evenly during the study period. Birds were observed at 8 different locations on the CSUEB campus (Figure 2) that had blooming flowers and shady and sunny microsites. Each location was observed for 45 min between 12:30 and 5:30 p.m. We conducted a total of 20 sessions across 8 locations on the CSUEB campus (sample sizes for location A = 3 sessions, B = 2, C = 4, D = 3, E = 2, F = 3, G = 2, H = 1) of these, 8 were categorized as hot sessions, and 12 were categorized as average. Sampling was uneven between sites because some sites stopped flowering while others began to flower. During sessions, we used scan sampling to record the behavior and microsite (sunny vs. shady) of every visible hummingbird within a 25 m radius at 5 min intervals. The following behavior categories were recorded: perching, preening, gaping, vocalizing, aggression, flying through, and foraging (fly‐catching or nectaring; Table 1). If a bird exhibited a combination of simultaneous behaviors, all simultaneously occurring behaviors were recorded (for example, perching and vocalizing). If a bird exhibited multiple sequential behaviors, only the behavior(s) that occurred at first sighting were recorded. During each session, the following environmental variables were recorded: cloud cover (sunny, partly cloudy, overcast), observation start and end times, the number of people that walked within 25 m of the feeders during the observations (low: < 10, moderate: 10–20, and high: >20), and the estimated number of open floral inflorescences within 25 m of the location.

**TABLE 1:** Descriptions of behaviors recorded during seminatural behavioral observation sessions.

Behavior	Description
Perching	Sitting on a perch
Foraging ‐ nectar	Making physical contact with a flower
Foraging ‐ insect	Fly‐catching or gleaning arthropods
Flying	Actively flying through the field of view but not hover‐feeding.
Preening	Moving own feathers with bill or foot
Vocalizing	Making a vocalization of any kind
Aggression	Chasing or directed physical contact with another bird

### 2.6 Data analysis

Feeder sessions and seminatural observations were categorized as hot or average based on whether the maximum temperature in the sun exceeded 35°C for over 50% of the trial. All statistical analyses were conducted in the software program R (R Core Team 2022). The effect of temperature on number of visits, visits per hour, and visit duration to each category of microsite was analyzed using general linear mixed models (GLMM) using the R package “lme4” (Bates et al., 2015). For feeder trials, candidate GLMMs were constructed with the total number of visits, average number of visits per hour (rounded to the nearest integer), or visit duration as response variables. Microsite and temperature were included as interacting fixed effects, and separate models were constructed using categorical (hot/average) versus maximum temperature in the sun as the temperature term. Julian date, session ID, and human presence were included as random effects. Session duration varied slightly between trials, so it was included as an offset term in all models. Total number of visits and visitation rate (visits/h) were modeled using a Poisson distribution. Visit duration data was heavily skewed to the right, and thus was centered and scaled by standard deviation before running models (Bolker et al., 2009; Meehan et al., 2020; Schielzeth, 2010). Initial data exploration showed the pollen count data were overdispersed, so an observation‐level random effect was added to all pollen deposition models to correct for overdispersion. Model selection was done for each test (response of total visits, visit rate, or visit duration and fixed effect of categorical or continuous temperature) by constructing models with every possible combination of fixed and random effects. The package ‘MuMIn’ was then used to select the best model (Bartoń, 2022). Candidate models were checked for normal distribution of residuals based on Q–Q and Shapiro–Wilk tests. Models were selected by the second order Akaike Information Criterion (AICc) (Bolker et al., 2009). Post‐hoc pairwise comparison for significant interaction terms was conducted using the package ‘emmeans’ with Bonferroni corrections (Lenth, 2022).

For seminatural observations, we constructed GLMMs with a Poisson distribution using the cumulative number of foraging visits to a flower as the response variable, the interaction of microsite (whether individual was observed visiting a flower in the shade or the sun) and ambient temperature (categorical or maximum temperature in the sun, as in feeder trial models) as the fixed effect, and a unique session ID, Julian date, human presence (using same scale as in feeder trials), location, and floral abundance as random effects. Observation session duration was included as an offset term. Model selection, quality control, and post‐hoc pairwise comparisons were conducted following the same process as in feeder trial models.

## 3 RESULTS

### 3.1 Feeder trials

We conducted a total of 30 feeder trials, during which we recorded 529 foraging visits by Anna's hummingbirds; 31% (*N* = 164) of all visits were in the shady microsite, while 69% (*N* = 365) were in the sunny microsite. Feeder trials were approximately balanced between hot (*N* = 14) and average (*N* = 16) ambient temperatures.

**FIGURE 4:**
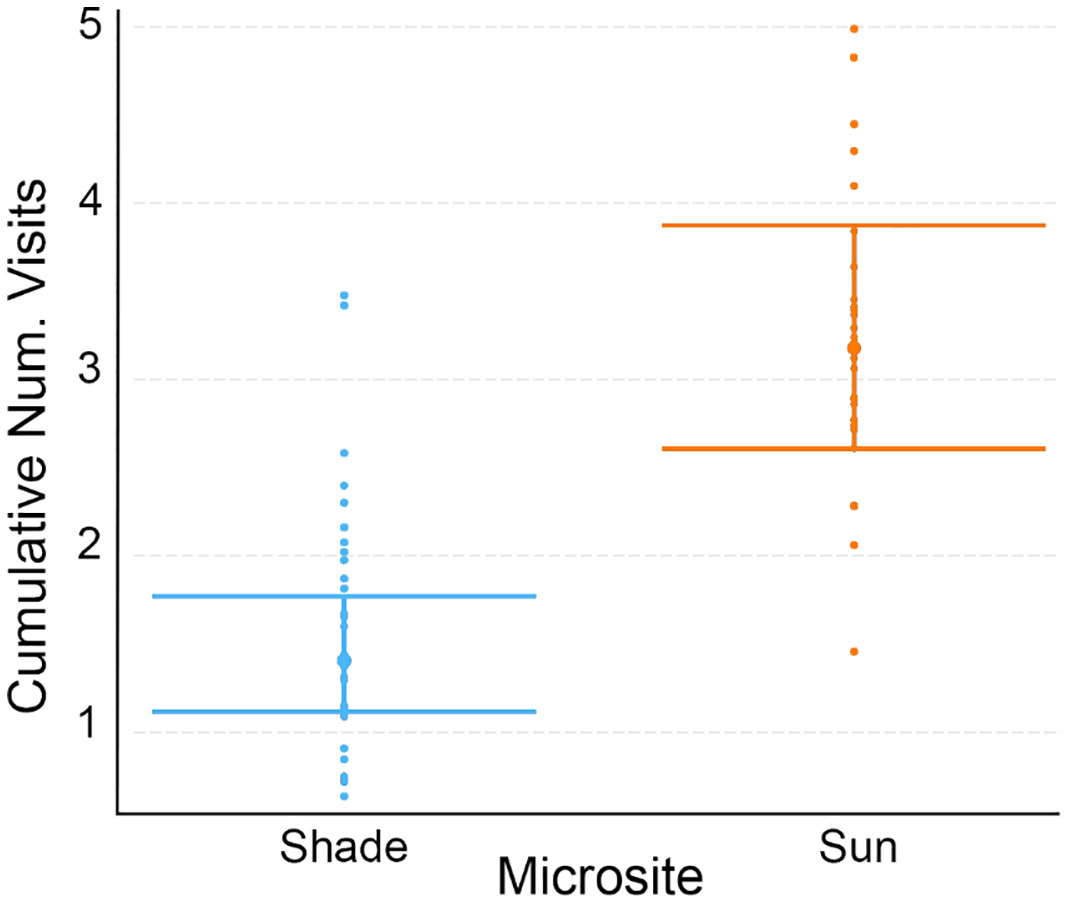
Predicted effects plot showing cumulative number of visits by Anna's hummingbirds per feeder trial in shade and sun microsites when maximum sun temperature is used as a fixed effect. Error bars are standard errors (SE).

The best model for cumulative number of visits included the interaction of microsite and maximum sun temperature as fixed effects, trial ID as the random effect, and an offset term for trial duration (Figure 4; Tables 2 and A1). We found a significant effect of microsite, but not ambient temperature or the interaction of ambient temperature and microsite. Sunny microsites had a significantly higher number of visits than shady microsites regardless of ambient temperature (Coef = 1.50, *z* = 2.83, *p* < .01). A model with the same random effects but with temperature as a categorical variable found a similar result (Tables 2 and A1), with sunny microsites receiving significantly more visits than shady microsites regardless of ambient temperature (Coef = 0.82, z = 4.85, *p* < .001).

**TABLE 2:** Final model for cumulative number of foraging visits in feeder trials with ambient temperature as the maximum temperature in the sun during the session and temperature as a categorical variable where “hot” is categorized as an ambient maximum temperature in the sun >35˚C and “average” is ≤35˚C.

Variable	Coefficient	SE	Lower 95% CI	Upper 95% CI	*z*‐value	*p*‐value
Model: Num. Visits ~ microsite*max_sun_temp + (1|session_id) + offset (session_duration)						
Intercept	−1.2	0.63	−2.43	0.03	−1.92	.06
Microsite (sun)	**1.5**	**0.53**	**0.46**	**2.25**	**2.83**	<.01**
Max. sun temp (˚C)	0.02	0.02	−0.02	0.05	0.89	.37
Microsite (sun)*Max. sun temp	−0.02	0.01	−0.05	0.01	−1.34	.18
Model: Num. Visits ~ microsite*temp_cat + (1|session_id) + offset (session_duration)						
Intercept	**−0.74**	**0.2**	**−1.12**	**−0.35**	**−3.75**	<.01*
Microsite (sun)	**0.82**	**0.17**	**0.49**	**1.15**	**4.85**	<.001**
Temp category (hot)	0.14	0.24	−0.34	0.61	0.57	.53
Microsite (sun)*Temp category (hot)	−0.02	0.2	−0.42	0.37	−0.11	.91

*Note*: Variables with significant effects are shown in bold.

*p < .05; **p < .01; ***p < .001.

**FIGURE 5:**
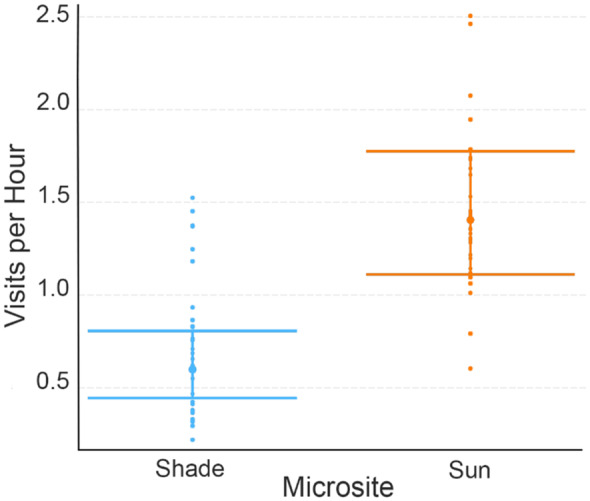
Effects plot showing number of visits per hour by Anna's hummingbirds per feeder trial in shade and sun microsites when maximum sun temperature is used as a fixed effect. Error bars are standard errors (SE).

When considering visitation rate as the dependent variable, the average visitation rate (visits/hour) was 2.67 ± 1.65 (SD) in shade and 5.75 ± 3.00 (SD) in sun during hot trials, and 2.02 ± 1.08 (SD) in shade and 4.91 ± 2.07 (SD) in sun during average trials. The best model for visitation rate included the interaction of microsite and maximum sun temperature as the fixed effect, trial ID as a random effect, and trial duration as an offset term (Figure 5, Tables 3 and A2). The only significant fixed effect was microsite, with sunny microsites receiving significantly more visits per hour than shady microsites regardless of ambient temperature (Coef = 1.76, *z* = 2.17, *p* < .05). An identical model with temperature as a categorical variable was the second best model during model selection, and had a similar result, with sunny microsites receiving significantly higher visitation rates regardless of temperature category (Tables 3 and A2; Coef = 0.96, *z* = 3.68, *p* < .001).

**TABLE 3:** Final model for visitation rate (visits/hour) in feeder trials with ambient temperature as the maximum temperature in the sun during the session and ambient temperature as a categorical variable where “hot” is categorized as an ambient maximum temperature in the sun >35˚C and “average” is ≤35˚C.

Variable	Coefficient	SE	Lower 95% CI	Upper 95% CI	*z*‐value	*p*‐value
Model: Visit rate ~ microsite*max_sun_temp + (1|session_id) + offset (session_duration)						
Intercept	**−2.19**	**0.83**	**−3.81**	**−0.06**	**−2.64**	<.01**
Microsite (sun)	**1.76**	**0.81**	**0.17**	**3.35**	**0.85**	<.05*
Max. sun temp (˚C)	0.02	0.02	−0.02	0.06	0.85	.40
Microsite (sun)*Max. sun temp	−0.03	0.02	−0.07	0.02	−1.17	.24
Model: Visit rate ~ microsite*temp_cat + (1|session_id) + offset (session_duration)						
Intercept	**−1.66**	**0.26**	**−2.18**	**−1.15**	**−6.35**	<.01**
Microsite (sun)	**0.96**	**0.26**	**0.45**	**1.47**	**3.68**	<.001***
Temp category (hot)	0.25	0.32	−0.37	0.87	0.8	.427
Microsite (sun)*Temp category (hot)	−0.18	0.31	−0.78	0.43	−0.57	.57

*Note*: Variables with significant effects are shown in bold.

*p < .05; **p < .01; ***p < .001.

**FIGURE 6:**
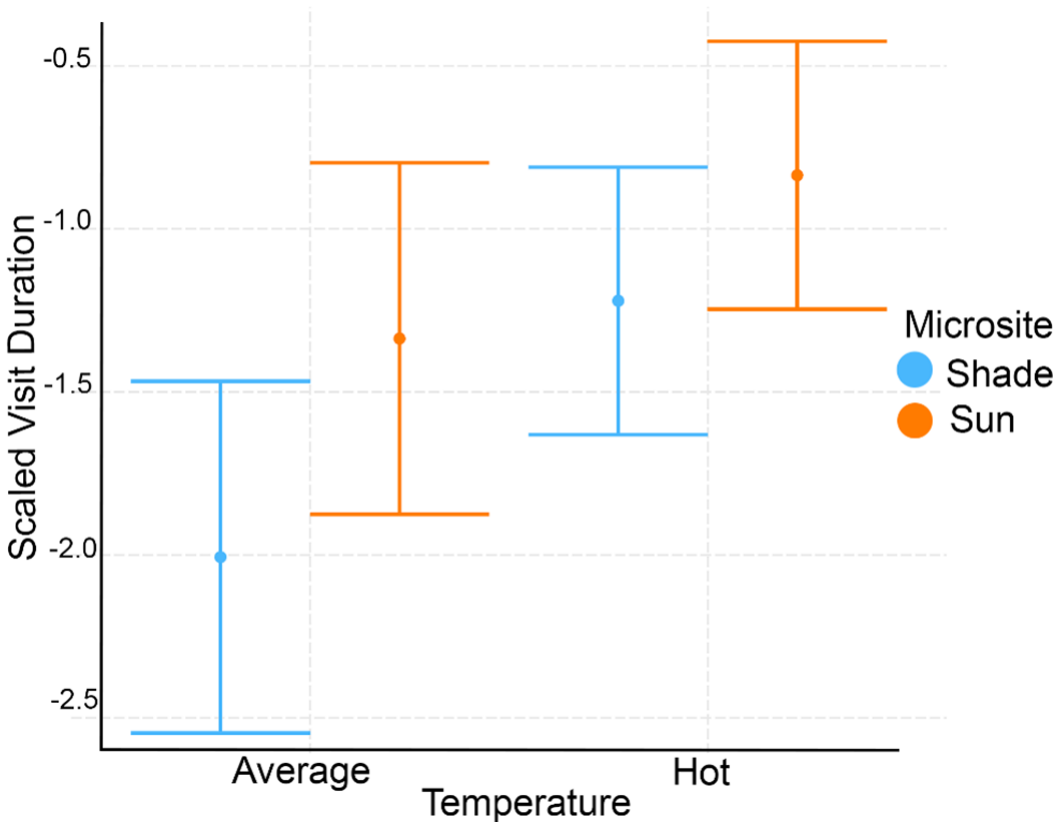
Categorical interaction plot showing average visit duration from feeder trials in sun and shade microsites on hot session and average sessions when temperature is a categorical variable (average vs. hot). Error bars are standard errors (SE).

When considering visit duration during feeder trials, the average visit duration in the shade was 41.41 s ± 25.34 (SD) and 52.85 s ± 12.79 (SD) in the sun during hot trials. The average visit duration in the shade was 28.81 s ± 18.23 (SD) and 45.61 s ± 14.93 (SD) in the sun during average days. The best model for the average visit duration included the interaction of microsite and categorical temperature as fixed effects, Julian day as a random effect, and trial duration as an offset term (Figure 6, Tables 4 and A3). No other models had a ΔAICc < 2. The final model found a significant effect of both temperature category and microsite, with higher visit duration on hot trials (Coef = 0.50, *t*‐value = 1.48, *p* < .05) and lower visit duration in the shade than in the sun (Coef = −0.67, *t*‐value = −2.22, *p* < .01).

**TABLE 4:** Final model for visit duration in feeder trials with temperature as a categorical variable where “hot” is categorized as an ambient maximum temperature in the sun >35˚C and “average” is ≤35˚C.

Model: Visit duration ~ microsite*temp_cat + (1|session_id) + offset (session_duration)						
Variable	Coefficient	SE	Lower 95% CI	Upper 95% CI	*t*‐value	*p*‐value
Intercept	**−2.34**	**0.27**	**−2.83**	**−1.81**	**−8.69**	<.01**
Microsite (shade)	**−0.67**	**0.3**	**−1.26**	**−0.08**	**−2.22**	<.01**
Temp category (hot)	0.5	0.34	−0.16	1.16	1.48	<.05
Microsite (shade)*Temp category (hot)	0.29	0.38	−0.46	1.03	0.75	.45

*Note*: Variables with significant effects are shown in bold.

*p < .05; **p < .01; ***p < .001.

### 3.2 Seminatural behavioral observations

**FIGURE 7:**
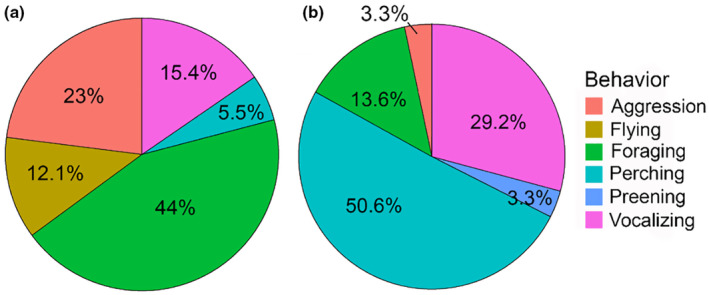
Time budgets of Anna's hummingbirds during hot seminatural behavioral observation sessions based on average percent of observations falling into each behavior category per session in (a) sunny and (b) shady microsites.

**FIGURE 8:**
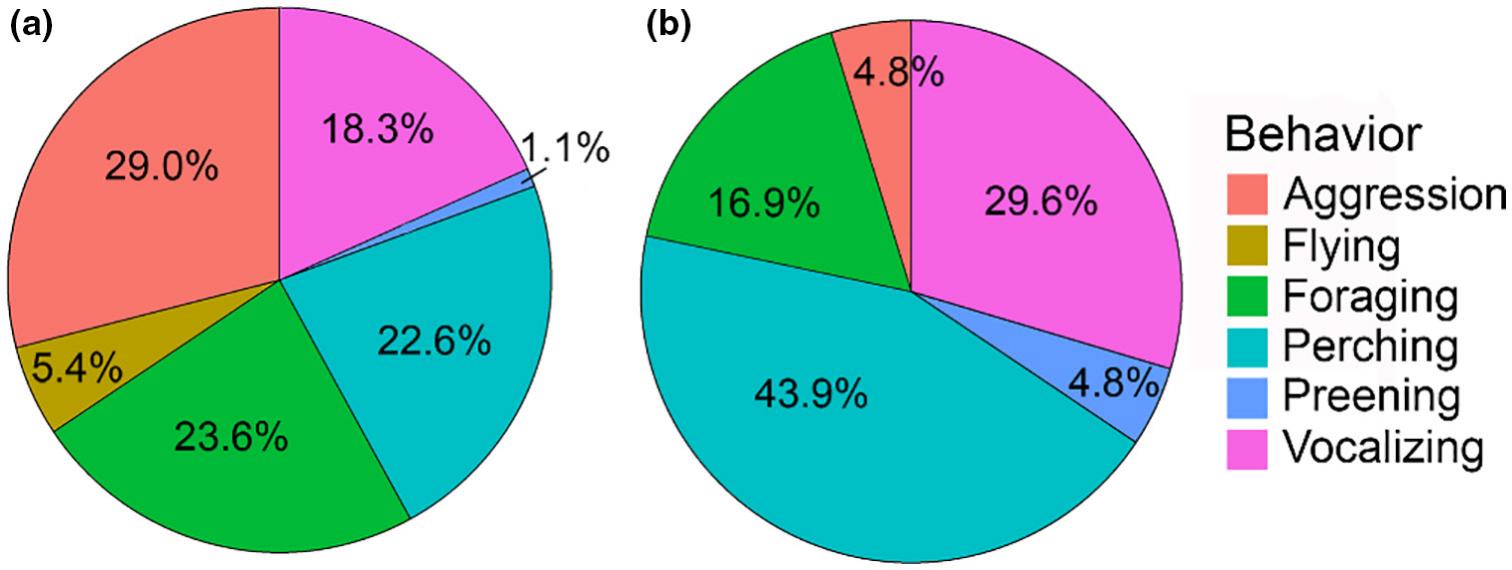
Time budgets of Anna's hummingbirds during average seminatural behavioral observation sessions based on average percent of observations falling into each behavior category per session in (a) sunny and (b) shady microsites.

We conducted a total of 20 sessions across 8 locations on the CSUEB campus (sample size per location: site A = 3 sessions, site B = 2, C = 4, D = 3, E = 2, F = 3, G = 2, H = 1); of these, 8 were categorized as hot sessions, and 12 were categorized as average. Out of a total of 409 individual observations of behavior across all sessions, 107 were of foraging. Session duration averaged 47 min ± 1.85 (SD), for a total of approximately 17 h of observations. Hummingbird time budgets were apparently different on hot and average days (Figure 7) with greater incidences of foraging, aggressive interactions, and flying on hot sessions as compared to average sessions. Birds spent less time perching, vocalizing (for example calling), and preening on hot sessions. Hummingbirds also apparently used microsites differently (Figure 8). Birds spent more time foraging, flying, and in aggressive interactions in sunny microsites, and more time perching, preening, and vocalizing in shady microsites. Birds were observed feeding at 9 different flowering ornamental plants during sessions (Table 5), two of which are native to California, of which Mexican sage (Lamiaceae, *Salvia leucantha*) and strawberry tree (Ericaceae, *Arbutus unedo*) were the most visited (Table A4).

**TABLE 5:** Final model from seminatural observations for cumulative number of visits (total number of birds observed foraging per session per microsite), with temperature as a categorical variable where “hot” is categorized as an ambient maximum temperature in the sun >35˚C and “average” is ≤35˚C.

Model: Semi‐natural observations total visits ~ microsite*temp_cat + (1|session_id) + offset (session_duration)						
Variable	Coefficient	SE	Lower 95% CI	Upper 95% CI	*z*‐value	*p*‐value
(Intercept)	**1.05**	**0.24**	**0.58**	**1.51**	**4.39**	<.001***
Microsite (sun)	−0.32	0.29	−0.89	0.24	−1.12	.26
Temp category (hot)	−0.22	0.37	−0.94	0.5	−0.6	.55
Microsite (sun)*Temp category (hot)	**1.15**	**0.4**	**0.36**	**1.94**	**2.87**	<.01**

*Note*: Variables with significant effects are shown in bold.

*p < .05; **p < .01; ***p < .001.

**FIGURE 9:**
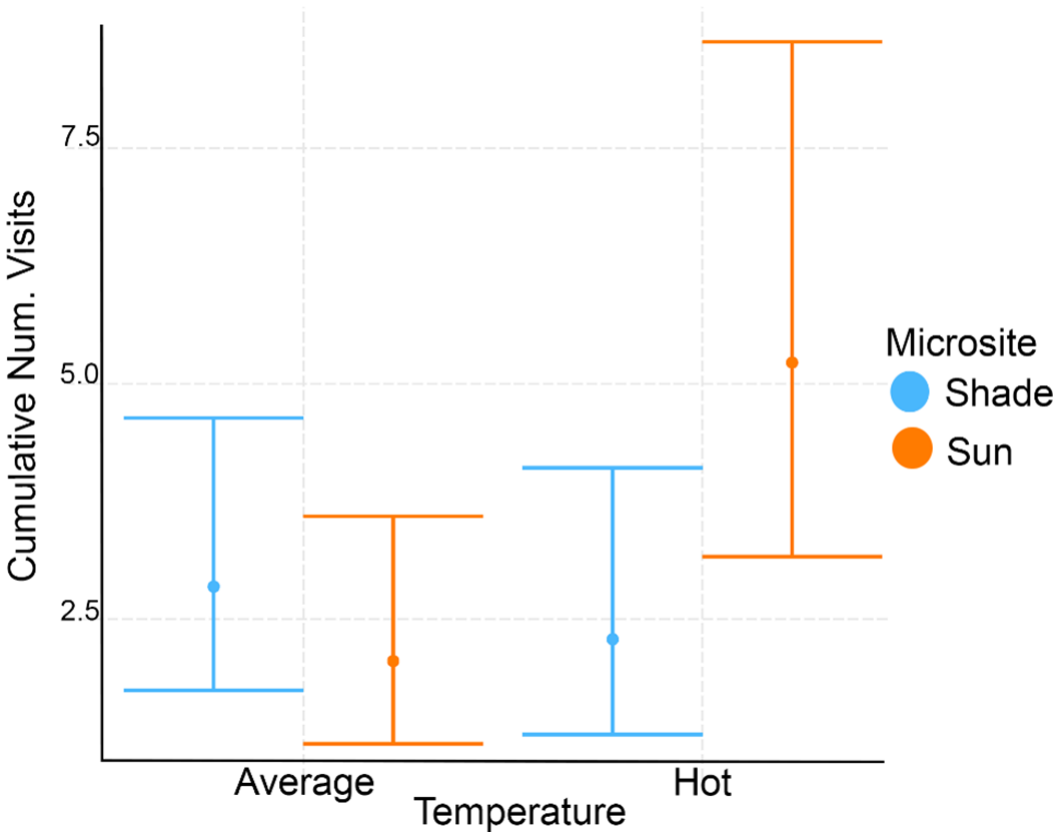
Categorical interaction plot showing the cumulative number of visits per seminatural behavioral observation session in sun and shade microsites on hot session and average sessions when temperature is a categorical variable (average vs. hot). Error bars are standard errors (SE).

The best model for the cumulative number of nectaring observations per session included the interaction of microsite and categorical temperature (hot vs. average) as the fixed effect and session ID and floral abundance as random effects (Figure 9, Tables 6 and A5). The interaction term was significant (Coef = 1.15, *z*‐value = 2.87, *p* < .01), and post‐hoc tests revealed that this was driven by differences in microsite use on hot sessions, where hot sessions had more foraging in the sun than in the shade (Ratio = 0.44, *z*‐ratio = −2.97, *p* < .05), a pattern which differed from their preferences on normal temperature days (Table A6).

**TABLE 6:** Ornamental plant species visited by hummingbirds during seminatural foraging observations from *N* = 20 observation sessions at 8 locations on the California State University East Bay (CSUEB) campus field sites.

Plant species	Plant family	Num. Visits shade	Num. Visits sun	Total num. Visits	Proportion of visits in sun
Salvia leucantha*	Lamiaceae	13	35	48	0.73
*Arbutus unedo*	Ericaceae	24	7	31	0.23
Gambelia speciosa*	Plantaginaceae	2	9	11	0.82
*Phormium cookianum*	Asphodelaceae	3	5	8	0.63
*Erythrina crista‐galli*	Fabaceae	0	2	2	1
*Lantana camara*	Verbenaceae	0	1	1	1
*Melaleuca viminalis*	Myrtaceae	0	1	1	1
*Eucalyptus* sp.	Myrtaceae	0	1	1	1
*Agapanthus praecox*	Amaryliidaceae	1	0	1	0

*Note*: An asterisk (*) indicates that the species is native to California.

### 3.3 Pollen deposition

**FIGURE 10:**
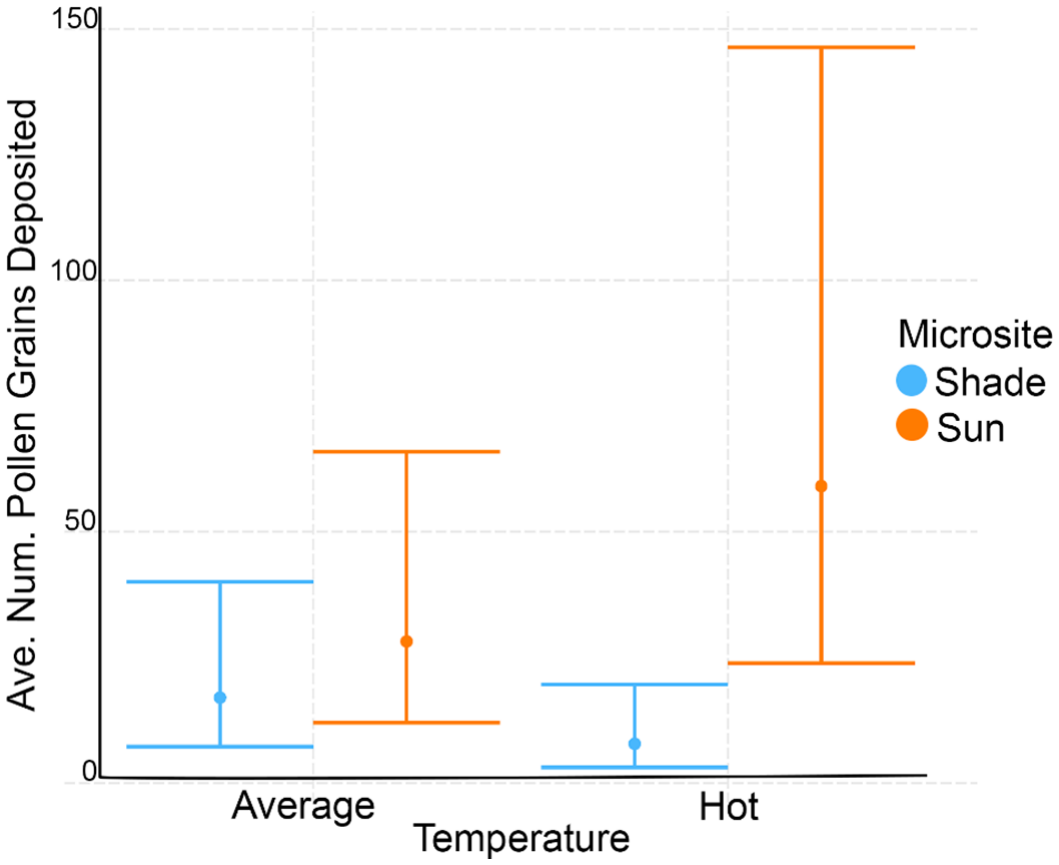
Categorical interaction plot showing average number of pollen grains deposited per feeder trial in sun and shade microsites on hot session and average sessions when temperature is a categorical variable (average vs. hot). Error bars are standard errors (SE).

When considering pollen deposition during the feeder trials, the average number of pollen grains deposited in the shade was 81.29 ± 157.80 (SD) and 593.02 ± 950.29 (SD) in the sun during hot trials. The average number of pollen grains deposited in the shade was 404.63 ± 747.27 (SD) and 275.38 ± 747.27 in the sun during average days. The average pollen load (total pollen grains deposited/total visits) in the shade was 14.90 ± 26.02 (SD) and 58.68 ± 109.22 (SD) in the sun during hot trials. The average pollen load in the shade was 80.37 ± 170.30 (SD) and 20.03 ± 29.25 (SD) in the sun during average days. The best model for average pollen deposition included the interaction of microsite and categorical temperature as fixed effects, Julian day and observation effect as random effects, and session duration as an offset term (Tables 6 and A6). We found the interaction term was very close to significant (Coef = 1.52, *z*‐value = 1.94, *p* = .05), but neither microsite nor categorical temperature was significant individually (Table 7). On hot trials the sunny microsite received much more pollen than the shady microsite, though the confidence interval is considerably wider for the sunny microsite than the shady microsite (Figure 10).

**TABLE 7:** Final model for pollen deposition on the false stigmas during feeder trials with temperature as a categorical variable where “hot” is categorized as an ambient maximum temperature in the sun >35˚C and “average” is ≤35˚C.

Model: pollen count ~ microsite*temp_cat + (1|julian_day) + (1|obs_effect) + offset (session_duration)						
Variable	Coefficient	SE	Lower 95% CI	Upper 95% CI	*z*‐value	*p*‐value
Intercept	**1.83**	**0.43**	**0.99**	**2.67**	**4.28**	<.001***
Microsite (sun)	0.51	0.54	−0.54	1.55	0.95	.34
Temp category (hot)	−0.78	0.63	−2.01	0.45	−1.24	.21
Microsite (sun)*Temp category (hot)	1.52	0.78	−0.02	3.06	1.94	.05

*Note*: Variables with significant effects are shown in bold.

*p < .05, **p < .01, ***p < .001.

## 4 DISCUSSION

The hypothesis that *Calypte anna* will forage preferentially in shady microsites on hot days was not supported by the feeder trials or seminatural behavioral observations. There were some apparent differences in how hummingbirds spent their time between different behaviors on hot sessions. The most frequently observed behavior in the sunny microsites during hot sessions was foraging (44%, Figure 5a), while the most frequently observed behavior in the sunny microsite during average sessions was aggression (29%; Figure 6b). Perching was the most common behavior in shady microsites during both hot and average temperature days (Figures 5 and 6), likely due to the inherent presence of perching sticks in shade. It may be that extremely hot ambient temperatures (the highest temperature recorded in the sun during our study was 48.57°C on July 9, 2021 around 1:37 p.m.) have some effect on the overall time budget of hummingbirds, but that this does not change their foraging microsite preferences due to the ways in which they detect food sources.

Previous research on microsite occupancy in other avian taxa has found that birds will preferentially forage in the shade during hot periods (Abdu et al., 2018; Cunningham et al., 2015; Lee et al., 2017; Van de Ven et al., 2019). In many taxa, birds will forage in the shade above certain operative temperatures even when there is a fitness cost to doing so (Cunningham et al., 2015; Van de Ven et al., 2019). In hummingbirds, there is evidence for context‐dependent and contrasting responses to ambient temperature. In territorial hummingbirds like *C. anna*, increased thermoregulatory costs can lead to either decreased foraging activity to minimize energy loss, or conversely, increased foraging activity to maximize energy gain (Powers et al., 2017; Shankar et al., 2019). In our study, it is possible that the increased thermoregulatory costs of high temperatures could be driving increased foraging, in combination with a preference for sunny microsites. However, Shankar et al. (2019) found that thermoregulation made up a small part of energy budgets when compared to activity costs, and Powers et al. (2017) point out that flight generates large quantities of extra heat, so this will need further study. The preference for sunny microsites may be based on the reliance of hummingbirds on visual cues for detecting both nectar sources and threats from predators (though cover could also hide them from predators). There is some evidence that birds may feel less able to leave an artificially shady microsite because of a shade cloth, leading to more vigilance and lower preference for these sites (Abdu et al., 2018). Hummingbird dependence on visual cues to locate flowers is well established, and flowers in the sun may look different to them than flowers in the shade due to their ability to see ultraviolet light (Stoddard et al., 2020).

Hummingbirds do not typically drink water because their water needs are generally met through the nectar in their diet (Clark & Russell, 2012; Nicolson & Fleming, 2003), which is usually somewhat dilute in ornithophilous flowers (Nicolson & Fleming, 2003). However, cooling through evaporative water loss is an important physiological thermoregulation mechanism for hummingbirds. At an average temperature of 24°C, over 30% of a hummingbird's required daily water volume is lost through evaporation, while at 40°C this can be up to 50% (Powers 1992). Gaping behavior allows birds to use evaporative cooling on hot days, and results in additional water loss. It is possible that hummingbirds need to forage for nectar more on hot days to keep up with their water needs. This increased demand for nectar sources on extremely hot days, combined with a preference for the visual stimuli presented by flowers in the sun, could explain the patterns we observed. While this study did not specifically quantify the frequency of gaping behavior, we did observe it incidentally throughout the study during hot trials and sessions, and future studies in this area should quantify this.

Our results demonstrate that contrary to our hypothesis, flowers in sunny microsites may experience increased pollen deposition on extremely hot days due to increased frequency and duration of hummingbird visits to flowers in sunny microsites on hot days. Similar to the trend observed in the seminatural observations, the apparent preference for foraging in sunny microsites was amplified on hot days, with the sunny microsite receiving much more pollen than the shady site on hot days or either site on average days. Plants in sunny microsites may actually see increased hummingbird pollination services both during extreme heat events and in the future under a warming climate regime; however, future studies should seek to directly quantify pollen deposition, pollen tube formation, seed set, and seed germination rates as a result of pollination during heat waves. Pollen deposition was lowest in the shady microsite on hot days, suggesting that hummingbird‐pollinated plants in shady microsites may be more likely to experience pollen limitation during extreme heat events. The study of how nectar properties may shift as a result of warming temperatures is an emerging area of research. For example, Shrestha et al. (2018) found that above a certain temperature, bees shift their preferences towards flower shapes and colors that stay cooler, and Russell and McFrederick (2022) found that higher temperatures can change nectar microbiomes, sugars, and bee foraging preferences. Pollinator behavior has major implications for plant conservation, particularly in plants that are specialists for hummingbirds and may not receive supplemental visits from invertebrate pollinators or other vertebrate pollinators.

It is thus not surprising that pollen deposition followed similar trends to visitation rate and visit duration, with sunny microsites on hot days receiving higher visitation and longer visit durations, and thus slightly higher pollen deposition. It is interesting that pollen deposition varied so much in sunny microsites on hot days; this is likely due to the fact that not all visitors had pollen already on their bodies when they visited the feeder. Thus, because there is a preference for sunny microsites on hot days, we see that there is greater variation in pollen deposition, as more birds are visiting overall.

Morgan et al. (2014) found evidence of individual foraging preferences in wild rufous hummingbirds (*Selasphorus rufus*). Future studies should control for individual hummingbirds by using color markings to distinguish between individuals if possible, though not all researchers are able to do this given welfare concerns to birds and variation in local regulations on auxiliary color markings (Tell et al. 2021). In addition, the sex and age of hummingbirds may affect their foraging decisions, as male, female, and juvenile hummingbirds in California have distinct diets (Hazlehurst et al., 2021). Another important factor that could have affected our results is aggressive territorial behavior, as being chased away from a feeder could artificially shorten visit duration or cause hesitancy to return. Previous research suggests that frequency of aggression declines at both low and high temperatures due to increased thermoregulatory costs (González‐Gómez et al., 2011). Future studies should also consider foraging behavior over the entire daytime period rather than just focusing on the hottest part of the day. It is possible that hummingbirds shift as much of their foraging behavior as possible to early morning to avoid afternoon heat, and thus there may be additional factors that could influence pollen deposition patterns on hot and average days. However, there is mixed evidence for this strategy in hummingbirds, perhaps due to their need to feed frequently throughout the day, unlike in many other avian taxa. Powers et al. (2017) found only one of five hummingbird species studied became inactive during hot periods when it lacked a thermal gradient for passive cooling, and overall temporal patterns of foraging activity seem to vary by hummingbird species, time of year, and location (Clark & Russell, 2012). It is not currently known if wild hummingbirds are facing declines due to increased temperatures, and more research is needed on this subject.

There has been extremely limited research in this area to date, but future studies should further explore how nectar properties and avian pollinator behavior may change in a warming climate. For example, future research could determine if hummingbird behavioral responses during extreme heat events vary depending on the nectar properties (such as sucrose concentrations) of plants. While this study was conducted in a seminatural environment that consisted primarily of cultivated plants, future studies should consider pollination in more natural environments, as patterns of preference and pollen deposition may differ in those habitats. This is especially relevant considering the potential effects of heat stress and increased evaporation during periods of extremely high temperatures on floral trait expression (Carroll et al., 2001).

## AUTHOR CONTRIBUTIONS

**Sabina Lawrence:** Formal analysis (lead); investigation (lead); methodology (equal); project administration (equal); validation (equal); visualization (equal); writing – original draft (equal); writing – review and editing (equal). **Jenny Hazlehurst:** Conceptualization (lead); formal analysis (equal); investigation (supporting); methodology (supporting); project administration (supporting); resources (lead); software (equal); supervision (lead); validation (equal); visualization (equal); writing – original draft (equal); writing – review and editing (equal).

## ACKNOWLEDGEMENT

The authors would like to thank the undergraduate research assistants who helped collect this data, especially Henry Odufalu. We also thank all of the members of the Pollination Ecology & Conservation lab, and members of the graduate thesis committee at CSUEB, including Dr. Erica Wildy and Dr. Nazzy Pakpour, whose early feedback helped shape this research.

## FUNDING INFORMATION

None.

## CONFLICT OF INTEREST STATEMENT

The authors have no conflict of interest to declare.

## Data Availability

The data that support the findings of this study are openly available in "Dryad" at https://doi.org/10.5061/dryad.x95x69ppd

## APPENDIX A

**TABLE A1:** Top ranked models from feeder trials for the cumulative number of visits by hummingbirds.

Rank	Intercept	Fixed effects					Fixed effects	Random effects	Offset	AICc	ΔAICc
		Microsite (sun)	Max sun temp	Temp category (hot)	Microsite (sun) * max sun temp	Microsite (sun) * temp category (hot)					
**1**	**−1.20** **SE = 0.63** ***p* = .06**	**1.50** **SE = 0.53** ***p* < .01**	**0.02** **SE = 0.02** ***p* = .37**	**na**	**−0.02** **SE = 0.01** ***p* = .18**	**na**	**Microsite * max sun temp**	**Session id**	**Session duration**	**373.5**	**0**
2	−1.28 SE = 0.62 *p* < .05	1.45 SE = 0.53 *p* < .01	0.02 SE = 0.02 *p* = .27	na	−0.02 SE = 0.01 *p* = .18	na	Microsite * maximum sun temperature	Session id, human presence	Session duration	374.4	1.43
3	−0.73 SE = 0.20 *p* < .001	0.82 SE = 0.17 *p* < .001	na	0.14 SE = 0.24 *p* = .57	na	−0.02 SE = 0.20 *p* = .91	Microsite * temperature category	Session id	Session duration	374.9	1.44
4	−0.75 SE = 0.24 *p* < .01	0.82 SE = 0.17 *p* < .001	na	0.24 SE = 0.24 *p* = .33	na	−0.02 SE = 0.20 *p* = .91	Microsite * temperature category	Session id, human presence	Session duration	375.1	2.13

*Note*: If “na” is present, that means a fixed effect was not present in that model. The model in bold was selected and is presented in the results. Only models that successfully converged and were not overdispersed are shown.

**p* < .05.

**TABLE A2:** Top ranked models from feeder trials for the visitation rate (visits/hour) by hummingbirds.

Rank	Intercept	Fixed effects					Fixed effects	Random effects	Offset	AICc	ΔAICc
		Microsite (sun)	Max sun temp	Temp category (hot)	Microsite (sun) * max sun temp	Microsite (sun) * temp category (hot)					
**1**	**−2.19** **SE = 0.83** ***p* < .01**	**1.76** **SE = 0.81 *p* < .05**	**0.02** **SE = 0.02 *p* = .40**	**na**	**−0.02** **SE = 0.02** ***p* = .24**	**na**	**Microsite * Max sun temp**	**Session id**	**Session duration**	**275.5**	**0**
2	−1.66 SE = 0.26 *p* < .001	0.96 SE = 0.26 *p* < .001	na	0.25 SE = 0.32 *p* = .43	na	−0.18 SE = 0.31 *p* = .57	Microsite * Temp category	Session id	Session duration	276.2	0.74
3	−2.26 SE = 0.82 *p* < .01	1.74 SE = 0.80 *p* = .03	0.02 SE = 0.02 *p* = .31	na	−0.02 SE = 0.02 *p* = .25	na	Microsite * Max sun temp	Session id, Human presence	Session duration	276.5	1.55
4	−1.69 SE = 0.30 *p* < .001	0.96 SE = 0.26 *p* < .001	na	0.36 SE = 0.31 *p* = .25	na	−0.18 SE = 0.31 *p* = .57	Microsite * Temp category	Session id, Human presence	Session duration	276.6	1.59

*Note*: If “na” is present, that means a fixed effect was not present in that model. The model in bold was selected and is presented in the results. Only models that successfully converged and were not overdispersed are shown.

**p* < .05.

**TABLE A3:** Top ranked models from feeder trials for the visit duration by hummingbirds.

Rank	Intercept	Fixed effects					Fixed effects	Random effects	Offset	AICc	ΔAICc
		Microsite (sun)	Max sun temp	Temp category (hot)	Microsite (sun) * max sun temp	Microsite (shade) * temp category (hot)					
**1**	**−2.34** **SE = 0.27** ***p* < .01**	**−0.67** **SE = 0.30** ***p* < .01**	**na**	**0.50** **SE = 0.34** ***p* = .02**	**na**	**0.29** **SE = 0.38** ***p* = 0.45**	**Microsite * Temp category**	**Julian day**	**Session duration**	**152.7**	**0**
2	−5.01 SE = 0.80	1.28 SE = 0.94 *p* < .01	0.07 SE = 0.02 *p* < .001	na	−0.02 SE = 0.03 *p* = .39	na	Microsite * Max sun temp	Julian day	Session duration	158.8	6.18

*Note*: If “na” is present, that means a fixed effect was not present in that model. The model in bold was selected and is presented in the results. Only models that successfully converged and were not overdispersed areshown.

**p* < .05.

**TABLE A4:** Top ranked models from seminatural observations for the cumulative number of visits by hummingbirds.

Rank	Intercept	Fixed effects			Fixed effects	Random effects	Offset	AICc	ΔAICc
		Microsite (sun)	Temp category (hot)	Microsite (sun) * temp category (hot)					
**1**	**1.05** **SE = 0.24** ***p* < .001**	**−0.32** **SE = 0.29** ***p* = .26**	**−0.22** **SE = 0.37** ***p* = .55**	**1.15** **SE = 0.40** ***p* < .01**	**Microsite * Temp category**	**Session id, Floral abundance**	**na**	**165.1**	**0**
2	1.13 SE = 0.29 *p* < .001	−0.32 SE = 0.29 *p* = .27	−0.35 SE = 0.44 *p* = .43	1.14 SE = 0.40 *p* < .01	Microsite * Temp category	Session id, Floral abundance, Julian day	na	166.7	2.75

*Note*: If “na” is present, that means a fixed effect was not present in that model. The model in bold was selected and is presented in the results. Only models that successfully converged and were not overdispersed are shown.

**p* < .05.

**TABLE A5:** Post‐hoc pairwise comparisons for significant interaction term from best model for seminatural observations cumulative visits by hummingbirds.

Contrast	Estimate	SE	*t* ratio	*p* value
Shade average/sun average	−0.67	0.3	−2.22	.21
Shade average/shade hot	−0.79	0.34	−2.33	.15
**Shade average/sun hot**	**−1.17**	**0.34**	**−3.47**	**<.01****
Sun average/shade hot	−0.17	0.34	−0.34	1
Sun average/sun hot	−0.5	0.34	−1.48	.87
Shade hot/sun hot	−0.39	0.23	−1.68	.63

*Note*: Significant effects are shown in bold.

*Note*: **p* < .05, ***p* < .01, ****p* < .001.

**TABLE A6:** Top ranked models from pollen deposition during feeder trials.

Rank	Intercept	Fixed effects			Fixed effects	Random effects	Offset	AICc	ΔAICc
		Microsite (sun)	Temp category (hot)	Microsite (sun) * temp category (hot)					
**1**	**1.83** **SE = 0.43** ***p* < .001**	**0.51** **SE = 0.54** ***p* = .34**	**−0.78** **SE = 0.63** ***p* = .21**	**1.52** **SE = 0.78** ***p* = .05**	**Microsite * Temp category**	**Julian day, Observation effect**	**Session duration**	**766**	**0**
**1**	**1.83** **SE = 0.43** ***p* < .001**	**0.51** **SE = 0.54** ***p* = .34**	**−0.78** **SE = 0.63** ***p* = .21**	**1.52** **SE = 0.78** ***p* = .05**	**Microsite * Temp category**	**Session id, Observation effect**	**Session duration**	**766**	**0**
2	4.12 SE = 0.43 *p* < .001	0.50 SE = 0.53 *p* = .35	−0.80 SE = 0.63 *p* = .21	1.52 SE = 0.78 *p* = .05	Microsite * Temp category	Julian day, Observation effect	na	766.4	0.4
2	4.11 SE = 0.43 *p* < .001	0.50 SE = 0.53 *p* = .35	−0.80 SE = 0.63 *p* = .21	1.52 SE = 0.78 *p* = .05	Microsite * Temp category	Session id, Observation effect	na	766.4	0.4
3	1.83 SE = 0.43 *p* < .001	0.51 SE = 0.60 *p* = .40	−0.78 SE = 0.63 *p* = .21	1.52 SE = 0.88 *p* = .09	Microsite * Temp category	Floral abundance, Observation effect	Session duration	767.3	1.37

*Note*: If “na” is present, that means a fixed effect was not present in that model. The model in bold was selected and is presented in the results. Only models that successfully converged and were not overdispersed are shown.

**p* < .05.
