# CORRIGENDUM

**DOI:** 10.1002/ece3.10214

**Published:** 2023-06-18

In the recent article by Lawrence and Hazlehurst (2023), the authors would like to add the photo credit to the Figure 1 caption as shown below.

**FIGURE 1** Anna’s hummingbirds (Calypte anna) visiting a hummingbird feeder hanging on a porch in summer. (Photo by Gina Christophersen).
